# Personal well-being and financial threats in Peruvian adults: The mediating role of financial well-being

**DOI:** 10.3389/fpsyg.2022.1084731

**Published:** 2023-01-27

**Authors:** Bertha Estela-Delgado, Gilmer Montenegro, Jimmy Paan, Wilter C. Morales-García, Ronald Castillo-Blanco, Liset Sairitupa-Sanchez, Jacksaint Saintila

**Affiliations:** ^1^Unidad de Administración, Escuela de Posgrado, Universidad Peruana Unión, Lima, Peru; ^2^Unidad de Salud Pública, Escuela de Posgrado, Universidad Peruana Unión, Lima, Peru; ^3^Escuela de Medicina Humana, Facultad de Ciencias de la Salud, Universidad Peruana Unión, Lima, Peru; ^4^Departamento de Gestión del Aprendizaje, Universidad del Pacífico, Lima, Peru; ^5^Escuela Profesional de Psicología, Facultad de Ciencias de la Salud, Universidad Peruana Unión, Lima, Peru; ^6^Escuela de Medicina Humana, Universidad Señor de Sipán, Chiclayo, Peru

**Keywords:** personal well-being, financial, threats, Peruvian adult, mediation

## Abstract

Crises negatively affect the economy of a country, increasing financial risk, as they affect work activities and the well-being of the population. This study aimed to examine the mediating role of financial well-being in the relationship between personal well-being and financial threats. A predictive cross-sectional study was conducted. The variables analyzed were personal well-being, financial threats, and financial well-being. A total of 416 Peruvian adults from the three regions of Peru participated. The mean age was M = 35.36, SD = 8.84, with a range of 19–62 years. To represent the statistical mediation model, a structural equation model (SEM) was used. The analysis showed that the variables were significantly related (*p* < 0.001). The theoretical model indicated a perfect mediation, also obtaining a good fit, *χ*^2^(168) = 394.3, CFI = 0.931, RMSEA = 0.057, SRMR = 0.062. The study showed that personal well-being serves as a basis for promoting financial well-being and this contributes to the reduction of financial threats.

## 1. Introduction

Health crises such as COVID-19 have led to unstable labor situations and increased labor concerns, negatively affecting the global and Peruvian economy (Barraza, 2020; Wilson et al., 2020; Durst et al., 2021). Financial risks have had an impact on labor and business activities, causing negative consequences due to temporary layoffs, business closures, and job insecurity (Alcover et al., 2020). The increase in unemployment was more constant along with the financial difficulties of micro and small businesses. Families were affected by economic uncertainty, expressing greater pessimism about the economic situation, and there was less financial well-being (Barrafrem et al., 2020). Informal workers are unable to earn income due to public health restrictions and depending on government assistance and food donations, these financial threats affect their financial well-being (Salameh et al., 2020; Botha et al., 2021). However, the more experience one has with economic hardship, the more financial threats to the population increase (Fiksenbaum et al., 2017b). Social scientists indicate that personal finances are related to well-being; therefore, financial crises predict poor physical and psychological health outcomes for the population (Richardson et al., 2013).

Financial well-being is considered as an objective condition when considering material economic resources, and it is also a subjective experience when considering and evaluating one’s own economic condition (Sorgente and Lanz, 2017; Kaur et al., 2021). Not all people have the same perception of their financial situation. Some people with few resources are satisfied with their lives, while others, full of opportunity and wealth, struggle because they don’t have enough finances (Grezo and Sarmany-Schuller, 2015). Financial well-being has been an important variable during the COVID-19 pandemic, due to the effect on occupational, economic, and health vulnerability during the quarantine period (Chori et al., 2021). Making good decisions has an impact on financial well-being, but you need to plan for the long term, saving, in order to establish short-term security (Fan, 2021). People often find it difficult to administer or manage their finances, which leads to behaviors that have a negative effect on their savings and increase their financial threats, as they become vulnerable to financial crises (Braunstein and Welch, 2002; Ullah and Yusheng, 2020). Studies show that adults lack financial knowledge and skills, as they are faced with many financial areas such as spending, savings, housing, retirement, and credit cards that could ensure their financial well-being, yet they possess low financial education that causes debts, savings, retirement plans to affect their future financial well-being (Rutherford and Fox, 2010; Sinha et al., 2018). In fact, being financially educated may help acquire attitudinal and behavioral roles related to financial well-being and alleviate or reduce the anxiety or stress that accompanies crises (Shim et al., 2009).

Personal well-being allows when the person faces challenges or threats, to go through a process of adaptation in order to balance demands such as psychosocial health and to have some coping strategies. This allows the person to emerge with adaptive resources for future challenges (Gonzalez et al., 2022). Whereas, constraints such as concerns about money or financial resources affect personal well-being (Rea et al., 2018). During the pandemic, parents have promoted well-being, health, and ability to cope with internal and external factors (Russell et al., 2020). Therefore, decreasing the factors that affect financial states allows for better financial well-being and improved personal well-being. Decreased personal well-being influences financial well-being due to crises and can have lasting effects on physical health, increased heart disease, lower job performance, and shorter life expectancy (Ferreira et al., 2021).

Financial problems contribute to an increase in negative psychosocial outcomes, such as psychological distress, depression, suicidal intent, and dissatisfaction with life, among others (Mamun et al., 2020). The financial threat is the way in which the person evaluates stressful situations and usually produces fear, worry, or uncertainty of financial stability and security, because as there is a financial crisis, financial situations also deteriorate (Marjanovic et al., 2015). It is important to make a primary assessment to establish the harm that some stressors may cause in the future. Therefore, the higher the estimation of harm, the higher the perception of the different stressors as threatening (Fiksenbaum et al., 2017b). The analysis of threat levels is followed by an evaluation of the potential aspects to address financial threats. Financial threats increase in times of crisis or financial deterioration (Folkman, 2013; Mamun et al., 2020).

### 1.1. Review of literature

#### 1.1.1. Financial well-being

Financial well-being is a person’s objective and subjective assessment of his or her current situation. A person’s dynamic assessment of his or her own well-being is determined by various personal and contextual factors that are changeable (Brüggen et al., 2017). Financial literacy has become an essential skill due to unstable global markets (Philippas and Avdoulas, 2019). Several studies have found that the influence of personal factors is important in financial well-being, since financial well-being allows control over finances and is able to absorb financial threats, the freedom to make decisions that promote the well-being of the individual (Zhang and Cao, 2010; Ponchio et al., 2019). Financial well-being results from meeting financial commitments, financial resilience for future events. In addition, behavioral factors such as spending restraint, active savings, and no borrowing for daily expenses, allow for greater financial well-being (Carton et al., 2022). Stress can lead to short-term credit decisions that can aggravate initial debt problems (Gathergood and Weber, 2014). Furthermore, the literature indicates that there is a detrimental relationship between financial hardship and mental health (Bialowolski et al., 2021). Thus, financial well-being can be a mediator between personal well-being and financial threats.

#### 1.1.2. Personal well-being

Subjective well-being is the cognitive appraisals of general satisfaction, emotional appraisals of happiness, and emotional balance (Diener et al., 1999; Diener and Oishi, 2009). Personal well-being has been evaluated in different ways such as life satisfaction, happiness, and general well-being, it also involves activities subject to finances, as there are a large number of factors that contribute to personal well-being. Personal well-being is a predictor of financial well-being (Gerrans et al., 2013). Models such as Joo (2008) indicate that financial well-being is a component of personal well-being. However, there is a causal link between financial well-being and personal well-being, since an increase in financial well-being is associated with an increase in personal well-being (Gerrans et al., 2013).

#### 1.1.3. Financial threats

**Figure 1:**
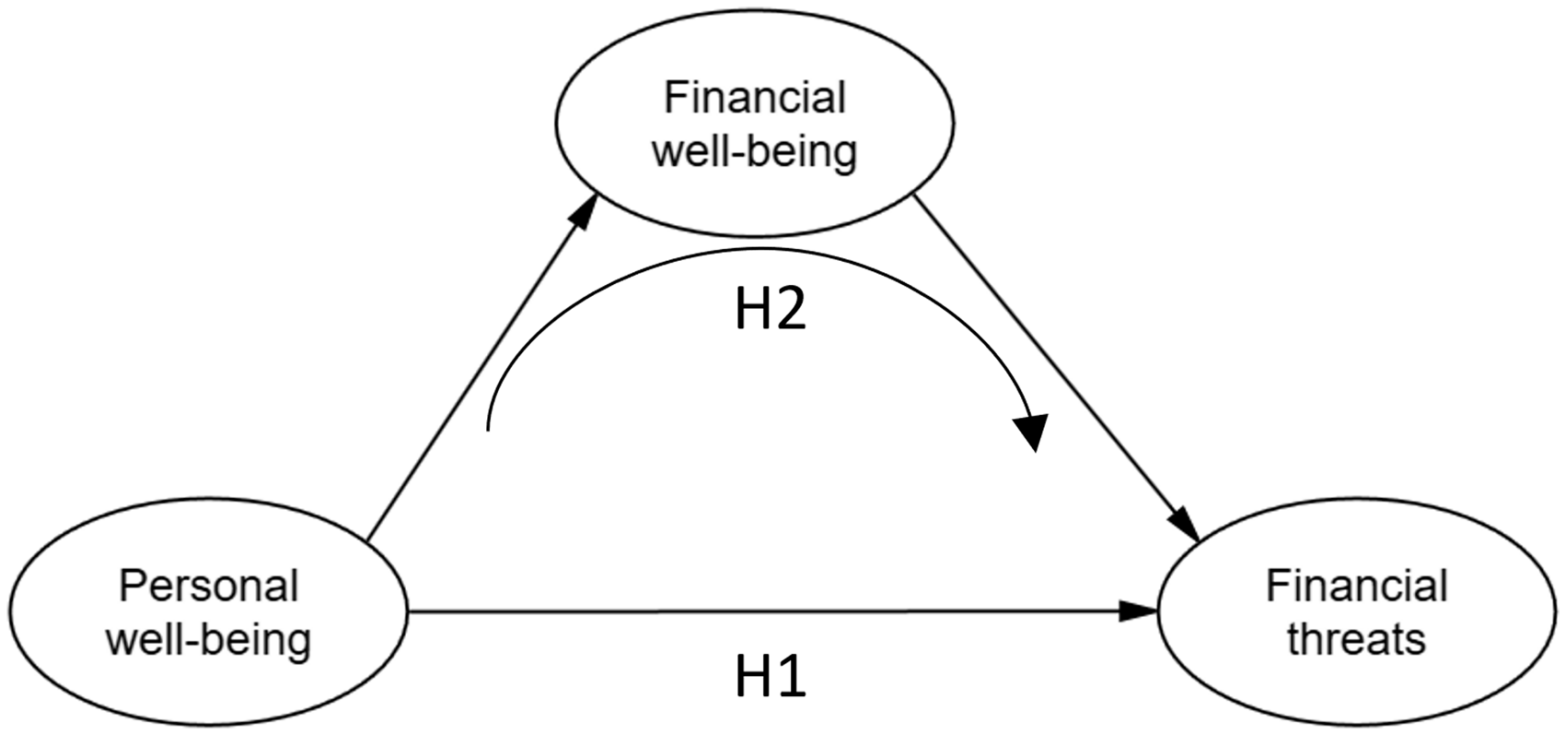
Theoretical model.

Financial threat refers to fearful-anxious uncertainty regarding current or future conditions. In the midst of economic crises, financial threat tends to increase more than normal. This to the likelihood of economic deterioration, high unemployment rates, and declining quality of life (Marjanovic et al., 2013; Fiksenbaum et al., 2017a). Likewise, Lazarus and Folkman (2013) indicate that threatening perceptions are not always based on reality, but more of a perceived danger of stress and focus on coping skills. In the face of this people whose financial well-being has been eroded their financial stability as a result of economic instability allows them to experience greater financial threat and leads to greater psychological distress. Likewise, people who experience greater financial threat are those who experience situations such as job loss, financial difficulties, loss of income, and stress (de Miquel et al., 2022; Figure 1).

Based on the above, it is proposed that financial well-being mediates the relationship between personal well-being and financial threats, considering the following hypotheses:

*Hypothesis 1*: Personal well-being will have an effect on financial threat.

*Hypothesis 2*: Financial well-being mediates the relationship between personal well-being and financial threat.

## 2. Materials and methods

### 2.1. Study design and population

A cross-sectional and explanatory study was designed considering latent variables represented by a system of structural equations (Ato et al., 2013). The number of participants was determined using Soper software that considers the number of observed and latent variables for structural equation models (SEM), whereby the anticipated effect size (λ = 0.3), statistical power levels (1 – β = 0.95), and the desired probability (α = 0.05), indicated a number of 184 of participants (Soper, 2021). The final sample consisted of 416 Peruvian adults from the 3 regions of Peru (coast, highlands, and jungle) using a convenience sampling method, taking into account the absence of data and lack of response.

### 2.2. Procedure

After approval by the ethics committee of the University (Cod: 2021- CE-EPG-000078), participants were invited to complete the questionnaire available from 21 November 2021, to 20 February 2022 *via* Google Forms, which allowed online sharing. Prior to data collection, the guidelines stipulated in the Declaration of Helsinki and the norms of confidentiality were considered by informing participants about the nature of the project, followed by obtaining informed consent. The completeness of the questionnaires presented below was evaluated.

### 2.3. Measurements

Measures of personal well-being, financial threat, and financial well-being adopted from existing research were considered and translated into Spanish according to established guidelines for the translation and cross-cultural validation of instruments (Tsang et al., 2017).

1. Initially, three PhDs with expertise in business administration and finance and accounting, fluent in English and Spanish, made a direct and independent translation of the three measures into Spanish (Peru)
2. Second, the first Spanish version was independently translated into English by two translators whose native language was English and who were fluent in Spanish
3. Third, based on both versions, the research team, together with the translators mentioned above, evaluated the translated versions and performed a comparative analysis with this existing version, considering some linguistic and cultural similarities. The items were evaluated by financial and management experts in the field who considered that the items were appropriate and that the instrument was relevant to the Peruvian population, so the initial version of the measures of personal well-being, financial threat, and financial well-being was developed
4. Fourth, a pilot test was conducted, in which the initial version was applied to 10 students to check the readability and comprehension of the items
5. In fifth place, the research group evaluated the pilot, test and no modifications were suggested, which made it possible to have the version of personal well-being, financial threat, and financial well-being (Annex 1). The instruments are described below.

#### 2.3.1. Financial well-being

Financial well-being was measured using the inventory that included six of the items from the Prawitz et al. (2012) measure of financial distress/financial well-being. Participants were asked to indicate their degree of financial stress on a scale of 1–10. For example, “on a scale of 1–10, where one is” overwhelmingly stressed “and ten is” no stress at all.” In this study, the model presented adequate reliability indices on the total scale (ordinal α = 092, ω = 0.89, *H* = 0.92), and the model presented adequate validity indices (*χ*^2^ = 85.732; *df* = 9; *p* = 0.000; CFI = 0.998, TLI = 0.997, RMSEA = 0.073, SRMR = 0.028).

#### 2.3.2. Personal well-being

Personal well-being will be measured using the PWI-A (International Wellbeing Group, 2006), which contained 8 items and a general well-being question that was used by the International Wellbeing Group to validate the index. Participants indicated their degree of satisfaction in different areas of life: life as a whole, standard of living, health, life achievements, personal relationships, present security, feeling part of a community, future security, and spirituality or religion. For each item, participants were asked to indicate a value from 0 (completely dissatisfied) to 10 (completely satisfied), with 5 being neutral. Cronbach’s alpha ranged from 0.70 to 0.85. In this study, 6 items were considered (see Appendix 1), the model presented adequate reliability indices (ordinal α = 0.90, ω = 0.92, *H* = 0.93), and the model presented adequate validity indices (*χ*^2^ = 73.556; *df* = 9; *p* = 0.000; CFI = 0.999, TLI = 0.998, RMSEA = 0.066, SRMR = 0.021).

#### 2.3.3. Financial threats

The five-element FTS was developed in accordance with existing threat measures and threat research (Marjanovic et al., 2013). The aim is to cover a wide range of the hypothetical financial threat construct with as few elements as possible. Its five items cover areas of uncertainty, risk, perceived threat (included to reinforce face validity), worry, and cognitive concern with current personal finances. The five items are supported along five-point scales, the endpoints of which change slightly to reflect the content of the item. A Cronbach’s alpha of 0.89 was obtained. In this study, the model presented adequate reliability indices (ordinal α = 0.98, ω = 0.87 and *H* = 0.96), and the model presented adequate validity indices (*χ*^2^ = 14.689; *df* = 2; *p* = 0.000; CFI = 0.999, TLI = 0.998, RMSEA = 0.064, SRMR = 0.020).

### 2.4. Data analysis procedure

The theoretical model under study was analyzed using structural equation modeling with the MLR estimator, which is appropriate for numerical variables and is robust to inferential normality deviations (Muthen and Muthen, 2017). The evaluation of the fit was performed with the comparative fit index (CFI), the root mean square error of approximation (RMSEA), and the standardized root mean square residual (SRMR). CFI values of >0.90 were used (Bentler, 1990), RMSEA <0.080 (MacCallum et al., 1996), and SRMR <0.080 (Browne and Cudeck, 1992). For the mediation analysis, the bootstrapping method was used with 5,000 iterations and a 95% confidence interval (Yzerbyt et al., 2018). Regarding reliability analysis, the internal consistency method was used with the alpha coefficient (α), ordinal α, and coefficient ω (McDonald, 1999; Hancock and Mueller, 2001; Pascual-Ferrá and Beatty, 2015) expecting magnitudes greater than 0.80 (Raykov and Hancock, 2005; Dominguez-Lara, 2016).

The structural equation modeling analysis was performed with the “R” software in version 4.0.5 and the “lavaan” library was used (Rosseel, 2012). The organization of the initial database and the first descriptive results were obtained with IBM SPSS Statistics 26 software.

## 3. Results

### 3.1. Sociodemographic

The final sample was comprised 416 Peruvian adults. The mean age was M = 35.36 (SD = 8.84) ranging from 19 to 62 years. Among them (Table 1), it is shown that the majority were between 29 and 38 years old (42.3%), with an income level of 0 to 930 (33.7%), from the coastal region (76.0%), with a high school technical education (32.7%), self-employed (50.7%), with an average financial education (57.9), and savings (56.3).

**Table 1:** Sociodemographic information.

Characteristics		*n*	%
Age	19–28	92	22.1
	29–38	176	42.3
	39–62	148	35.6
Sex	Female	269	64.7
	Male	147	35.3
Level of income	5,001 or more	13	3.1
	2,500–5,000	50	12.0
	1,501–2,500	88	21.2
	931–1,500	125	30.0
	0–930	140	33.7
Region of origin	Coast	48	11.5
	Jungle	316	76.0
	Sierra	52	12.5
Level of education	Graduate	118	28.4
	None	10	2.4
	Postgraduate	27	6.5
	Primary	19	4.6
	Secondary	106	25.5
	Technical Bachelor’s Degree	136	32.7
Work modality	Dependent	205	49.3
	Independent	211	50.7
Financial education	Under	63	15.1
	Elevated	9	2.2
	Below average	69	16.6
	Above average	34	8.2
	Average	241	57.9
Savings	No	182	43.8
	Yes	234	56.3

### 3.2. Preliminary analysis

The scores of the study variables were scaled between values between 0 and 30 in order to facilitate their reading. Table 2 shows the correlation matrix and the descriptive results, where the correlation results are between 0.24 and 0.50 in absolute value. In addition, this table also shows the internal consistencies that were found between the values of 0.87 and 0.94.

**Table 2:** Descriptive statistics, internal consistencies, and correlations for the study variables.

Variables	*M*	*SD*	A	α	1	2	3
1. Financial well-being	18.2	5.4	−0.1	0.88	-		
2. Personal well-being	22.5	4.9	−0.5	0.94	0.50***	-	
3. Financial threats	27.6	9.3	0.1	0.87	−0.36***	−0.24***	-

M = Mean, SD = Standard Deviation and A = Skewness, α = Cronbach’s Alpha. ***p < 0.001.

### 3.3. Structural model

**Figure 2:**
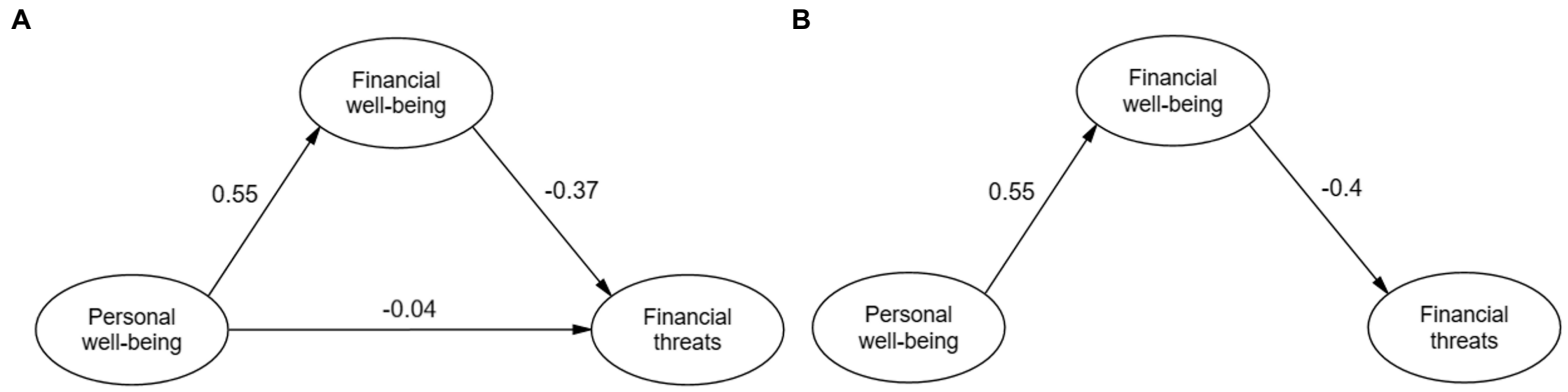
Results of the structural model: **(A)** Including direct effect and **(B)** excluding direct effect.

In the theoretical model analysis, an adequate fit was obtained, 2 (167) = 393.4, *p* < 0.001, CFI = 0.931, RMSEA = 0.057, SRMR = 0.057, results that can be visualized in the left model in Figure 2 (model a). Given the close to null value of the effect of personal well-being on financial threats, and in consideration of the parsimony criteria of the model proposal, we chose to restrict this relationship to zero, also obtaining a good fit, 2 (168) = 394.3, *p* < 0.001, CFI = 0.931, RMSEA = 0.057, SRMR = 0.062. This model is presented on the right side of the Figure 2 (model b).

For the mediation analysis, bootstrapping of 5,000 iterations was used. Then, with respect to *H*2, the mediating effect of financial well-being on the effect of Personal Well-Being on Financial Threats is confirmed, *β* = −0.22, *p* < 0.001, 95% CI [−0.12, −0.05].

## 4. Discussion

The financial crises resulting from the COVID-19 pandemic have resulted in considerable losses not only in the health aspect but also in the economy in Peru and worldwide. Peru had been suffering economic problems and these were aggravated by the various measures adopted by the government, where many companies had to close and there was a massive layoff of personnel, which increased unemployment (Barreto et al., 2021). Subsequently, due to the normalization of economic activities and the new measures taken by the government, the economy recovered after a downturn; however, the drop in formal employment and loss of income continue without a visible recovery (Barreto et al., 2021). The purpose of this study was to examine how personal well-being influences financial threats through financial well-being. The results supported that personal well-being was positively associated with financial well-being, which, in turn, was negatively associated with financial threats. The study contributes to a more complete understanding of the process of personal well-being through the underlying mechanism of how personal well-being affects financial threats during a crisis.

As expected, personal well-being was positively associated with financial well-being. The result was consistent with previous studies (Nanda and Banerjee, 2021), as they indicate a healthy balance between savings and expenses crucial to personal and financial well-being (Brüggen et al., 2017). So financial well-being allows for a state of overall happiness or satisfaction with financial situations, and encompasses greater security with income or savings and thus maintaining material security (Mahdzan et al., 2020). Thus, proper financial behavior and self-control allow for greater financial well-being (Strömbäck et al., 2017). Therefore, credit counseling can help in having positive financial behaviors, which results in better health and greater financial well-being (O’Neill et al., 2013).

Likewise, the results indicated that financial well-being was negatively associated with financial threat. Previous studies indicate that the results are consistent, since financial well-being indicates an adequate standard of living without financial disruption or instability, while in times of financial crisis or threat, it tends to increase and may be higher than normal due to economic deterioration (Marjanovic et al., 2013). This may also be due to the pessimistic economic outlook people have about the future. Thus, those who assess their own economic situation in comparison with the national or global situation have better ways of coping with financial threats. Unlike pessimistic people who are less prepared for negative economic shocks, the negative effect of financial threats is increased (Barrafrem et al., 2020).

Finally, our results indicated that financial well-being mediated the relationship between personal well-being and financial threats. This indicates that those with decreased personal well-being report greater economic problems and increased financial distress (Fiksenbaum et al., 2017a). The study also showed that there is no indirect relationship between personal well-being and financial threats. This suggests that financial well-being mediates individual well-being resources and financial threats due to the fact that the individual could change his or her behavior in the midst of financial crises by reducing spending and increasing income, seeking employment (de Miquel et al., 2022). Therefore, financial well-being has a function in which it allows the evaluation of personal resources for stress management. However, more studies are needed to extend the assessment of coping and motivations in adverse situations to reduce financial and psychological distress.

Generally, people often make decisions to improve their state and well-being financially. These decisions include spending responsibly, opening savings accounts, and borrowing in order to grow assets and protect financial resources (Sehrawat et al., 2021). However, in the current national and global financial context, financial decisions can be particularly challenging. On the one hand, at the national level, the political crisis that the country (Peru) is going through is challenging for the economic life of Peruvian households, considering that financial well-being depends to a certain extent on political stability and people’s confidence in the government (Barrafrem et al., 2021). In fact, trust or distrust in public institutions is an important pillar to face financial challenges and improve financial well-being (Algan and Cahuc, 2010). On the other hand, at the global level, there is a concern on the part of political leaders to find effective strategies to improve the financial sector, financial well-being, and stability of households (World Bank, 2013). Whereas citizens can easily find themselves trapped in an unfavorable economic situation if not managed with responsible financial behavior, therefore, it is imperative to adopt financial measures such as identifying personality traits, improving financial literacy or specific economic behaviors through appropriate financial education to help people cope in these times of difficult economic and health crises to ensure their financial well-being (Netemeyer et al., 2018; Riitsalu and Murakas, 2019). Moreover, to increase or improve financial well-being, it is necessary to adopt adequate financial strategies that lead to an increase in satisfaction in relation to the financial situation; these strategies include having defined financial objectives in such a way that they help to have a systematic saving for future emergencies, having control over income and expenses through a budget, not generating unnecessary debts, and making expenses according to the financial possibilities that are available; all of these are fundamental to increase financial well-being (Netemeyer et al., 2018; Riitsalu and Murakas, 2019).

### 4.1. Implications

The results may be useful to organizations or professionals capable of formulating public policies. Variables such as financial well-being and threats make it possible to develop financial education programs that have an impact on families affected by crises. Because better management of financial affairs could help to control, improve financial skills and decisions, and achieve financial and personal well-being. Likewise, programs should raise awareness among teachers and children in order to motivate them to save from childhood, so that as adults they can increase their level of confidence and make better financial decisions. More cross-cultural studies are needed due to the scarcity of studies and the performance of studies with other sociodemographic characteristics that allow greater representativeness of the adult population. Thus, financial phenomena such as well-being and other variables with different characteristics and dynamics could be tested.

### 4.2. Limitations

This study has some limitations to consider. First, the study was cross-sectional and cannot adequately explore the causal relationships between variables, hence a more robust analysis. Therefore, longitudinal studies are recommended to test for causal connections. Second, political, social, and economic factors may be different in other countries, which may lead to different results. Third, the self-administered instruments used together may not represent a measure of an individual’s financial behavior and threat.

## 5. Conclusion

Personal well-being provides greater overall satisfaction with financial matters, in turn, appropriate financial behavior results in greater financial well-being. Financial well-being provides a standard of living with greater financial stability and makes it possible to cope with financial crises. So lower financial well-being can cause greater financial problems due to the inability to cope with financial threats. Thus, when a person perceives greater well-being, it serves as a basis for promoting financial well-being and contributes to the reduction of financial threats.

## Data availability statement

The data on which this study is based can be requested from the corresponding author.

## Ethics statement

The studies involving human participants were reviewed and approved by the study was reviewed by the ethics committee of the Universidad Peruana Unión (Cod: 2021-CE-EPG-000078). The patients/participants provided their written informed consent to participate in this study.

## Author contributions

BE-D, GM, JP, and WM-G participated in the conceptualization. WM-G, JS, and LS-S were in charge of the methodology and software. WM-G and RC-B performed validation, formal analysis, and research and commissioned data and resource conservation. First draft writing, review, and editing, visualization, and supervision were handled by WM-G, RC-B, JS, and LS-S. All authors contributed to the article and approved the submitted version.

## Conflict of interest

The authors declare that the research was conducted in the absence of any commercial or financial relationships that could be construed as a potential conflict of interest.

## Publisher’s note

All claims expressed in this article are solely those of the authors and do not necessarily represent those of their affiliated organizations, or those of the publisher, the editors and the reviewers. Any product that may be evaluated in this article, or claim that may be made by its manufacturer, is not guaranteed or endorsed by the publisher.
