# Supplementary material for: Personal well-being and financial threats in Peruvian adults: The mediating role of financial well-being
Source: Front Psychol. 2023 Jan 27;13:1084731. doi: 10.3389/fpsyg.2022.1084731 (PMC9912842; doi:10.3389/fpsyg.2022.1084731)
Supplement: Supplementary file 1 [file Presentation_1.pdf]

Annex 1

Scales in English/ Spanish

The following questions ask how satisfied you feel, on a scale from 0 to 10. Zero means you feel completely dissatisfied. Ten means you feel completely satisfied. The middle of the scale is 5, which means you feel neutral; neither satisfied nor dissatisfied.

Las siguientes preguntas preguntan qué tan satisfecho se siente, en una escala de 0 a 10. Cero significa que se siente completamente insatisfecho. Diez significa que te sientes completamente satisfecho. El medio de la escala es 5, lo que significa que te sientes neutral; ni satisfecho ni insatisfecho.

Financial Wellbeing/ Bienestar financiero

<table>
<tr><th colspan="5">Financial Wellbeing</th><th colspan="5">Bienestar financiero</th></tr>
<tr><td colspan="5">1. On a scale of 1 to 10 where one is ‘‘overwhelmingly stressed’’ and ten is ‘‘no stress at all,’’ what do you feel is the level of your financial stress today?</td><td colspan="5">1. En una escala del 1 al 10, donde uno está "abrumadoramente estresado" y diez es "sin estrés en absoluto", ¿cuál cree que es el nivel de su estrés financiero hoy?</td></tr>
<tr><td>1</td><td>2</td><td>3</td><td>4</td><td>5</td><td>6</td><td>7</td><td>8</td><td>9</td><td>10</td></tr>
<tr><td colspan="5">2. On a scale of 1 to 10 where one is ‘‘completely dissatisfied’’ and ten is ‘‘completely satisfied,’’ how satisfied are you with your present financial situation?</td><td colspan="5">2. En una escala del 1 al 10, en la que uno está "completamente insatisfecho" y diez está "completamente satisfecho", ¿qué tan satisfecho está con su situación financiera actual?</td></tr>
<tr><td>1</td><td>2</td><td>3</td><td>4</td><td>5</td><td>6</td><td>7</td><td>8</td><td>9</td><td>10</td></tr>
<tr><td colspan="5">3. On a scale of 1 to 10 where one is ‘‘feel completely overwhelmed’’ and ten is ‘‘feel very comfortable,’’ how do you feel about your current financial situation?</td><td colspan="5">3. En una escala del 1 al 10, donde uno es " sentirse completamente abrumado "y diez es" "sentirse muy cómodo" ", ¿cómo se siente con respecto a su situación financiera actual?</td></tr>
<tr><td>1</td><td>2</td><td>3</td><td>4</td><td>5</td><td>6</td><td>7</td><td>8</td><td>9</td><td>10</td></tr>
<tr><td colspan="5">4. On a scale of 1 to 10 where one is ‘‘worry all the time’’ and ten is ‘‘never worry,’’ how often do you worry about being able to meet normal monthly living expenses?</td><td colspan="5">4. En una escala del 1 al 10, donde uno es "preocuparse todo el tiempo" y diez es "nunca se preocupe", ¿con qué frecuencia se preocupa por poder cubrir los gastos de vida mensuales normales?</td></tr>
<tr><td>1</td><td>2</td><td>3</td><td>4</td><td>5</td><td>6</td><td>7</td><td>8</td><td>9</td><td>10</td></tr>
<tr><td colspan="5">5. On a scale of 1 to 10 where one is ‘‘no confidence’’ and ten is ‘‘high confidence,’’ how confident are you that you could find the money to pay for a financial emergency that costs about twice your weekly income?</td><td colspan="5">5. En una escala del 1 al 10 donde uno es "falta de confianza" y diez es "alta confianza", ¿qué tan seguro está de que podría encontrar el dinero para pagar una emergencia financiera que cuesta</td></tr>
</table>

					aproximadamente el doble de sus ingresos semanales?				
1	2	3	4	5	6	7	8	9	10
6. On a scale of 1 to 10 where one is ‘‘all the time’’ and ten is ‘‘never,’’ how frequently do you find yourself just getting by financially and living from payslip to payslip?					*6. En una escala del 1 al 10, en la que uno es "todo el tiempo" y diez es "nunca", ¿con qué frecuencia se las arregla económicamente y vive con un sueldo?*				
1	2	3	4	5	6	7	8	9	10

Personal Wellbeing / *Bienestar personal*

**Personal Wellbeing**					***Bienestar personal***				
1. Thinking about your own life and personal circumstances, how satisfied are you with your life as a whole?					*1. Pensando en su propia vida y circunstancias personales, ¿qué tan satisfecho está con su vida como un todo?*				
1	2	3	4	5	6	7	8	9	10
2. How satisfied are you with your standard of living?					*2. ¿Qué tan satisfecho está con su nivel de vida?*				
1	2	3	4	5	6	7	8	9	10
3. How satisfied are you with your health?					*3. ¿Qué tan satisfecho estás con tu salud?*				
1	2	3	4	5	6	7	8	9	10
4. How satisfied are you with what you are achieving in life?					*4. ¿Qué tan satisfecho está con lo que está logrando en la vida?*				
1	2	3	4	5	6	7	8	9	10
5. How satisfied are you with your personal relationships?					*5. ¿Qué tan satisfecho está con sus relaciones personales?*				
1	2	3	4	5	6	7	8	9	10
6. How satisfied are you with how safe you feel?					*6. ¿Qué tan satisfecha estás con lo segura que te sientes?*				
1	2	3	4	5	6	7	8	9	10
7. How satisfied are you with feeling part of your community?					*7. ¿Qué tan satisfecho está con sentirse parte de su comunidad?*				
1	2	3	4	5	6	7	8	9	10
8. How satisfied are you with your future security?					8. ¿Qué tan satisfecho está con su seguridad futura?				
1	2	3	4	5	6	7	8	9	10

9. How satisfied are you with your spirituality or religion?					*9. ¿Qué tan satisfecho está con su espiritualidad o religión?*				
1	2	3	4	5	6	7	8	9	10

Financial Threat /Amenaza financiera

Please indicate how you feel about your current financial situation by answering the following questions.

Indique cómo se siente acerca de su situación financiera actual respondiendo las siguientes preguntas.

**Financial Threat**	***Amenaza financiera***
1. How uncertain do you feel?	*1. ¿Qué tan inseguro se siente?*
1 = Not At All to 5 = Extremely Uncertain	*1 = Nada a 5 = muy inseguro*
2. How much do you feel at risk?	*2. ¿Cuánto riesgo siente?*
1 = Not At All to 5 = A Great Deal	*1 = Para nada a 5 = Mucho*
3. How much do you feel threatened?	*3. ¿Cuán amenazado se siente?*
3. How much do you feel threatened?	*1 = Nada a 5 = muy amenazado*
4. How much do you worry about it?	*4. ¿Cuánto te preocupa?*
1 = Not At All to 5 = A Great Deal	*1 = Para nada a 5 = Mucho*
5. How much do you think about it?	*5. ¿Cuánto piensas en ello?*
1 = Not At All to 5 = A Great Deal	*1 = Para nada a 5 = Mucho*
